# Anti-Interleukins for Severe Coronavirus Disease-2019: Hype or Hope?

**DOI:** 10.31138/mjr.33.4.384

**Published:** 2022-12-31

**Authors:** Athina Dimosiari, Dimitrios Patoulias, Demosthenes Makris

**Affiliations:** 1 Department of Internal Medicine, European Interbalkan Medical Centre, Thessaloniki, Greece,; 2 Department of Intensive Care Medicine, University Hospital of Larissa, Faculty of Medicine, University of Thessaly, Larissa, Greece

**Keywords:** COVID-19, interleukin-1, interleukin-6, canakinumab, anakinra, sarilumab

In late December 2019, in Wuhan, Hubei Province, China, a novel coronavirus, named severe acute respiratory distress syndrome coronavirus-2 (SARS-CoV-2), emerged. Most infected patients developed atypical pneumonia, while the majority of cases were primarily linked to the Huanan Seafood Wholesale Market. Mean incubation period was calculated to be 5.2 days.^1,2^ Epidemic rapidly spread worldwide, and the World Health Organization (WHO) declared the SARS-CoV-2 outbreak to be a public health emergency of international concern on 31 January 2020. The WHO finally characterised the disease as a “pandemic” on March 11, 2020, since WHO authorities stated that they are “deeply concerned both by the alarming levels of spread and severity and by the alarming levels of inaction”.^1,3^ To date, SARS-CoV-2 has affected individuals in almost every country all over the world. Three years later, as of November 2, 2022, approximately 631 million subjects have been infected worldwide, with almost 6.6 million documented deaths (coronavirus disease-2019 [COVID-19] Dashboard by the Centre for Systems Science and Engineering at Johns Hopkins University).

A significant improvement in the understanding of the pathogenesis of COVID-19 has been noted during the last 2 years, however the disease burden remains high.^1,4^ Severe COVID-19 is characterized by systemic hyper-inflammation, cytokine storm and rapid progression to respiratory failure and acute respiratory distress syndrome (ARDS). Major inflammatory cytokines, such as interleukin (IL)-6, IL-1, IL-8 and tumour necrosis factor alpha (TNF-α), have been shown to be predictors of disease severity and mortality, therefore, it was relatively early proposed that they should represent both prognostic biomarkers, but also treatment targets in COVID-19.^1,5,6^ Immunothrombosis, triggered by neutrophils and monocytes and resulting in the formation of microthrombi in small vessels, finally leading to thrombosis (thrombo-inflammation) and disseminated intravascular coagulation, is another key mechanism implicated into COVID-19 related complications, and therefore represents a reasonable treatment target.^7^

Cytokine release syndrome (CRS) is a systemic inflammatory response, first described in the early ‘90s. Various infections and certain drug classes can provoke such a response, like T-cell-engaging immunotherapies, utilised for the treatment of haematologic malignancies.^1,8^ Underlying pathophysiology remains unclear, with a massive release of a number of pro- and anti-inflammatory cytokines mediated by immune and non-immune cells. IL-6 might be the most crucial pro-inflammatory cytokine, as its’ release induces a pleiotropic response, activating cellular and innate immunity, as well as T-helper (Th)-2 and Th17 cells differentiation. Additionally, it stands out as the initial component of cytokine storm in COVID-19, as it binds to soluble IL-6 receptor (sIL-6R), attaching to almost any human cell and inducing vascular growth factor (VGF) production, along with secretion of more pro-inflammatory cytokines (such as IL-1).^1,9^ Earlier during this process, IL-1 induces gene expression and cytokine release in macrophages and dendritic cells, taking part into both non-specific and specific immunity, and provoking the continuous secretion of a pro-inflammatory complex that results in systemic inflammatory response syndrome. This metabolic cellular derangement represents the major underlying aetiology of septic shock and initiation of ARDS, gastrointestinal and neurological disorders, all being hazardous during COVID-19 course, supporting the hypothesis that the inhibition of IL-1 could be a promising therapeutic target to prevent hemodynamic changes and systemic organ inflammation and dysfunction.^1,10,11^

Thus, a vivid and ongoing discussion regarding the role of antirheumatic drugs targeting various stages of the inflammatory cascade in COVID-19 has started,^12^ by means of improvement in surrogate endpoints.

Concerning anakinra, data initially retrieved from observational studies suggested that this IL-1 receptor antagonist might produce a significant decrease in the odds for COVID-19 related death by 68%, while it might also decrease the odds for invasive mechanical ventilation by 62%, compared to standard of care, regardless of comorbidities or PO2:FiO2 ratio at baseline.^13,14^ Similarly, data regarding the use of canakinumab, a monoclonal antibody targeting IL-1β, was limited and scarce, indicating a rather favourable effect on COVID-19 surrogate outcomes.^15^

However, a recently published Cochrane meta-analysis of all relevant randomised controlled trials (RCTs) with IL-1 blockers in COVID-19 failed to demonstrate any significant effect either with anakinra or with canakinumab.^16^ Neither anakinra nor canakinumab exerted a significant effect on COVID-19 mortality, while none of these agents produced clinical improvement.^16^ A recently published RCT also failed to document any treatment benefit with anakinra among subjects hospitalised due to severe COVID-19.^17^ Similarly, in the formerly published DAWn-Antico study, anakinra treatment in patients with severe COVID-19 and hyper-inflammation did not result in significant improvement in efficacy outcomes, including COVID-19 related mortality.^18^

On the other hand, CRS in adults has been formerly successfully treated with IL-6 receptor inhibitors (tocilizumab and sarilumab) and IL-6 inhibitors (siltuximab); hence, RCTs were designed to examine their effectiveness and safety in severe COVID-19. In a prospective meta-analysis by the WHO in 2021 in a total of 10,930 patients, it has been demonstrated that initiation of IL-6 antagonists correlated with a significant decrease in the odds for COVID-19 death by 14%, compared to standard of care or placebo.^19^ Additionally, it was shown that IL-6 antagonists decreased the odds for the composite endpoint of invasive mechanical ventilation or death due to COVID-19 by 23%, without increasing the risk for secondary bacterial infections or any other serious adverse events.^19^ These results were confirmed by another recent meta-analysis of relevant RCTs, demonstrating a significant decrease in the risk for death by 25% and in the risk for intubation by 24%, with utilization of IL-6 antagonists compared to standard of care.^20^ In addition, patients allocated to IL-6 antagonist treatment experienced a significant increase in the odds for hospital discharge.^20^ Of note, recent data retrieved from a RCT conducted in Greece enrolling adults with severe COVID-19 and pO_2_:FiO_2_ ratio <200 mm Hg documented that tocilizumab is not inferior to baricitinib, a Janus kinase inhibitor approved for use in severe COVID-19, across a number of “hard” efficacy endpoints.^21^

Collectively, at present, it seems that IL-6 antagonists are superior to IL-1 blockers across a number of COVID-19 outcomes. Current evidence supports the use of IL-6 antagonists in therapeutic algorithms for the treatment of severe COVID-19, while IL-1 blockers seem that they do not exert beneficial effects on major outcomes, despite the initial enthusiasm. However, it is still unclear if these agents have similar efficacy against newer emerging SARS-CoV-2 variants, and of course, if there are any differences in their efficacy and safety according to prior vaccination status. Future trials are awaited to shed further light on these observations. Head-to-head comparison of these drug classes could definitely provide more certain answers. Treatment efficacy and safety of combined immunomodulation, including interleukin antagonists, should also be assessed in forthcoming RCTs, since observational data suggest the superiority of such combinations for the treatment of severe COVID-19.^22,23^
